# Improved Atomization via a Mechanical Atomizer with Optimal Geometric Parameters and an Air-Assisted Component

**DOI:** 10.3390/mi11060584

**Published:** 2020-06-11

**Authors:** Inna Levitsky, Dorith Tavor

**Affiliations:** 1 Department of Chemical Engineering, Shamoon College of Engineering, PO Box 950, Beer-Sheva 84100, Israel; 2 Green Processes Center, Shamoon College of Engineering, PO Box 950, Beer-Sheva 84100, Israel

**Keywords:** mechanical atomizer, air-assisted atomizer, swirl chamber, drop diameters, air/liquid mass flow ratio

## Abstract

Atomization of liquid media is a key aim in various technological disciplines, and solutions that improve spray performance, while decreasing energy consumption, are in great demand. That concept is very important in the development of liquid fuel spray atomizers in high-efficiency microturbines and other generator systems with low inlet pressure and a wide range of power supply. Here we present a study of the liquid atomization characteristics for a new mechanical atomizer that has optimal geometric parameters and a preliminary swirl stage. In our air-assisted atomizer, air is introduced through a swirl chamber positioned at the exit of the mechanical atomizer. The optimized mechanical atomizer alone can achieve *D_32_* drop diameters in the range of 80 to 40 µm at water supply pressures of 2 to 5 bar, respectively. The addition of an air swirl chamber substantially decreases drop sizes. At an air–liquid ratio (ALR) equal to 1, water pressures of 2.5 to 3 bar and air supply pressures 0.35 to 1 bar, *D*_32_ drops with diameters of 20–30 µm were obtained. In an air-assisted atomizer the parameters of the mechanical atomizer have a much stronger influence on drop diameters than do characteristics of the air-swirl chamber. Using a mechanical atomizer with optimal geometrical dimensions allows limiting the liquid supply pressure to 5 bar; but when an air-assisted component is introduced we can recommend an ALR ≈ 1 and an air supply pressure of up to 1 bar.

## 1. Introduction

Atomization of liquid media is an essential process in many different industries, so solving the technological challenges it presents is of significance for a wide range of applications. Efforts to improve the atomization process are being made in order to increase combustion efficiency in internal combustion gasoline and diesel engines [1,2], to reduce the cost of air humidification and cooling in hothouses [3,4,5], to aid in air purification [6,7], to improve firefighting appliances while minimizing water usage [8], and to achieve better product solidification for food industries [9]; liquid atomization is also used in the production of pharmaceuticals [10], in the treatments of plants and field for agriculture [11], in textile and tobacco-processing plants [12], and many others.

The atomization of the liquid is accomplished via various atomizer designs. The current designs for liquid atomization may be broadly classed into the following groups:

- Centrifugal nozzles, in which the working medium is given a tangential velocity component and exits through an outlet coaxial with the swirl chamber [13,14].
- Nozzles with impediments, in which the working medium discharges from the nozzle under high pressure and interacts with an obstacle installed in front of the nozzle [15,16]. Both of the above atomizer types use only the working pressure of the medium transformed into flow velocity to break the liquid up into small-sized particles. Such atomizers are described as *mechanical*.
- Atomizers that use gaseous media (air, steam, gas) to atomize the liquid. Such atomizers are of the air-assisted (two-phase) type [17,18,19].

The main benefit of pressure-swirl atomizers is their high energy-efficiency, and many industrial processes use them because of their combination of reliability, ability to achieve droplets of small dimensions, and high performance [20]. In many widely used pressure swirl atomizers, a liquid enters the swirl chamber through a number of tangential holes or slots. Centrifugal force causes the liquid to spread within the chamber as a hollow conical spray, with spray angles ranging from 30° to almost 180° depending on the application. Atomization occurs not only because of the break-up of the liquid sheet but also because of collisions between droplets and the interaction between droplets and air [21,22,23]. The finest atomization occurs at high pressures and at wide spray angles [24]. Atomizer performance has been found to be related to physical and experimental properties such as surface tension, viscosity, mass flow rate, density and injection pressure [25,26,27,28]. The mean diameter of drops is strongly associated with the injection pressure. With an increase in the pressure-drop across the atomizer, the liquid exits from the nozzle with a greater velocity, which then creates more intense disturbances on the liquid surface, increasing the quality of atomization. The effect of varying the injection pressure is usually more visible at low injection pressures than at high injection pressures [29]. Rashad et al. found that increasing the injection pressure from 8 bar to 12 bar decreases the *D*_32_ drop diameters from 69 µm to 55 µm [30]. However, if increasing pressure from 2 to 10 bar reduces the drop diameters by 45%, then an increase from 10 to 20 bar reduces the drop diameters by 28% and from 20 to 90 bar the reduction rate is just 42% [31]. It has been found that there exists a critical injection pressure of 15 bar beyond which the spray angle and drop sizes become practically independent of the pressure [32]. 

The geometrical parameters of the atomizer, such as the exit diameter of the swirl chamber (*d_n_*) and its length (*l_0_*), the swirl chamber diameter (*D_s_*) and its length (*L_s_*), the atomizer characteristic (*A*) and inlet tangential cross sectional area (*F_t_*), have direct effects on the swirling motion inside the swirl chamber and consequently are characteristics of the atomization process [33,34]. Chen et al. studied the influences on drop sizes of the ratio of length to swirl chamber diameter (*L_s_/D_s_*) the number of feed slots, the injection pressure, and the liquid’s viscosity, and concluded that an increase in the *L_s_/D_s_* ratio and liquid viscosity decreases the swirling motion [35]. The relation *L_s_/D_s_* should be low to minimize any friction loss. However, the swirl chamber should be designed with a proper height to separate the streams flowing out through the liquid inlet ports. For many designs, an *L_s_/D_s_* ratio ranging from 0.5 to 1.0 is assumed, although it was suggested that higher values of *L_s_/D_s_*, up to 2.75, might improve atomization [36]. Observations have indicated that the *D*_32_ decreases discernibly and continuously with decreasing *l_0_*/*d_n_*, with the effect on *D*_32_ in the range of 0.4–2.82 µm [37]. The drop diameters also diminished with a reduction of the inlet port area (*F_t_*) [38]. The length of the tangential channels (*l_t_*) of the swirl chamber also affects the characteristics of the atomizer. Short inlet channels are not effective and, if the channel is not long enough, the flow cannot be tangential, and will instead deflect to the axis of the swirl chamber. This deflection will decrease the momentum, increase the discharge coefficient, decrease the spray angle and diminish the quality of atomization. It may be concluded that beginning from *l_t_*/*d_t_ >* 2, the value of the discharge coefficient and spray angle remains constant, where *d_t_* is the diameter of tangential channels [39]. 

Another geometrical parameter is the convergence angle α of the swirl chamber. Considerable research findings indicate that the convergence angle has an inverse effect on performance parameters, with film thickness and discharge coefficient increasing with the increase of angle α. Increasing the angle of the swirl chamber to the nozzle from 60° to 90° increases the nozzle discharge coefficient from 0.3 to 0.35. That said, in cases when the spray angle is smallest and the film thickness is largest at a convergence angle of 90°, this geometry might be preferred as it is easier and less expensive to manufacture compared to a geometry with a smaller convergence angle [40]. 

The atomization of liquid by a mechanical atomizer can be improved by introducing a swirl-air component. Air-assisted atomizers have many advantages due to their ability to work at relatively low fuel supply pressures and to produce finer spraying. Rizkalla and Lefebvre reported that *D*_32_ becomes smaller with the increase of air velocity, air/liquid ratio, and air density [41]. Fraser et al. found that the air-to-liquid mass flow ratio has little effect on *D*_32_ for ratios exceeding 1.5 [42]. Those investigations describe a two-phase atomizer, in which after the pressurized liquid exits from the chamber the air is delivered as spray through the radial channels. When the vane angle is 45°, it was shown that the air-pressure drop has little effect on droplet size, if the drop in pressure exceeds 0.02 bar. However, the droplet diameter decreases drastically when the air pressure drop grows from 0 to 0.02 bar [43]. An analysis of the operational parameters of two-phase atomizers shows that the air supply pressure is 2 bar and above. For example, the atomizer of the JS company operates at 2.2 bar, that of the PNR company at a range of 2–4 bar, that of the Lechler company at 2 bar, that of Armstrong at 9.3 bar, and that of Optiquide Ltd between 6 and 8 bar. It should be noted that the compressed air supply pressure has the main effect on the energy consumption of the two-phase atomizers. Development of an atomizer for liquid fuel spray that will operate in a wide range of flow rates and low inlet pressure will reduce the energy consumption in microturbines and other generators.

The current work is a study of our mechanical atomizer with optimized geometric parameters, conducted as the first stage of the research, followed by an investigation of the utility of adding to the system an air supply through a swirl chamber having tangential channels of various diameters. The effect of the interaction of the liquid spray with the swirling air is investigated while changing the various operational and geometrical characteristics of the atomizer. 

## 2. Materials and Methods

### 2.1. Mechanical Atomizer 

**Figure 1:**
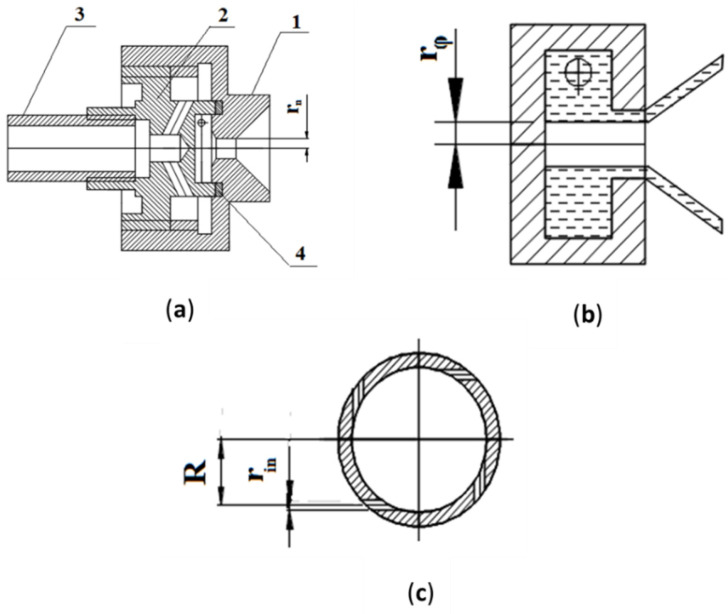
Mechanical atomizer (**a**) Atomizer assembly: 1—body, 2—swirl chamber, 3—adapter, 4—o-ring; (**b**) flow structure; (**c**) swirl chamber geometry.

The mechanical atomizer design is presented in Figure 1a. The atomizer is comprised of a swirl chamber with tangential ducts made in separate bush {2}. The bush is installed in the body {1} with the output nozzle of the chamber sealed from the inlet cavity by an o-ring {4} and compressed by the bush {2}. The water is supplied to the atomizer through a nosepiece {3}.

The characteristics of the liquid sheet emanating from the atomizer depend on the geometrical parameters of its swirl chamber. These can be expressed in dimensionless form as atomizer characteristic *A*, and defined as [39]:

(1) $$A=\frac{R\cdot r_{n}}{n\cdot r_{in}^{2}}$$

where *r_n_* is the radius of the discharge nozzle (Figure 1a), *R* is the swirl radius, *r_in_* is the radius of the tangential inlets (Figure 1c), and *n* is their number. The characteristic *A* is usually defined as the ratio of the axial flux of angular momentum to the axial linear momentum flux, which in this case is closely approximated by *A = U_t_/U_ax_*, where *U_t_* and *U_ax_* are the liquid tangential and axial velocities at the nozzle exit. According to the theory of the centrifugal nozzle, the impact of characteristic *A* on the velocities’ ratio is considerable up to the values of *A* = 2.5–4, after which the rise in *A* has little effect on the liquid’s flow rate coefficient and spray angle [39]. Increasing it further leads to rising hydrodynamic losses in the swirl chamber due to the friction of the flow against the end surfaces, which also lowers the ratio between the tangential and axial velocities, increases the flow rate coefficient, and diminishes the spray angle. Hence, for an available value of inlet pressure, the geometry of the above-listed parameters must be set so as to provide for the highest value of tangential velocity, while reducing the diverse hydrodynamic losses. In view of the above, the geometrical parameters of the experimental atomizer were optimized at the following values: *R* = 2 mm, *L_s_* = 1.5 mm, *r_n_* = 0.4 mm, *r_in_* = 0.25 mm, *n* = 4, *A* = 3.2. This geometry was adopted in order to provide a water flow rate of *m_w_* ≈ 2.0 × 10^−3^–2.5 × 10^−3^ kg/s with a supply pressure of 2 bar. In the atomizer we developed, *L_s_/D_s_* was equal to 0.3, *l_0_/d_n_* = 2, *l_t_/d_t_* = 2, and the convergence angle α of the swirl chamber was equal to 90°. 

### 2.2. Air-Assisted Atomizer 

**Figure 2:**
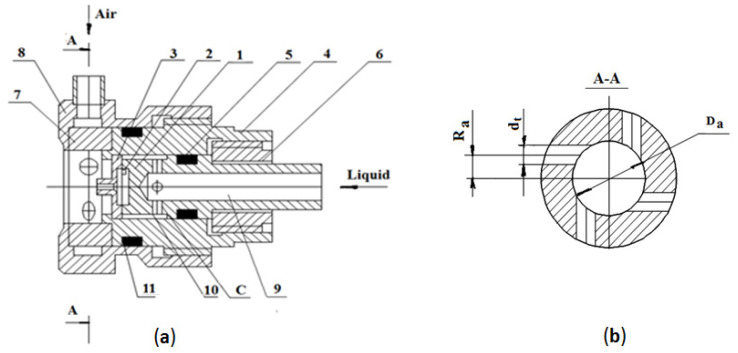
Design of an air-assisted atomizer. (**a**) Atomizer assembly: 1—swirl chamber, 2—grooves, 3—nozzle, 4—body, 5, 11—sealing ring, 6—nut, 7—bush, 8—adapter, 9—tip, 10—radial channels. (**b**) Cross section of the air swirl chamber.

The design of the air-assisted atomizer is shown in Figure 2. The atomizer consists of a mechanical atomizer with a swirl chamber {1} and bush {7}, and with tangential channels for introducing air at the exit of the mechanical atomizer. Water enters the swirl chamber from collector *C* through the tip {9} and the radial channels {10}.

This design, with the output channel of the mechanical atomizer positioned as a separate part {3}, is meant to create the possibility of changing the diameter of the swirl chamber outlet nozzle so as to study the effect of various channel diameters on spray quality. The swirl chamber has a nozzle {3} pressed to the edge of the body {4} by nut {6}, with the cavity sealed with a ring {5}. Body {4} holds in place the swirl chamber {7} installed in the adapter {8}. The swirl chamber {7} is of an open design and contains four tangential channels (Figure 2b). The region for installing the swirl-air chamber {7} is sealed with a ring {11}. 

**Figure 3:**
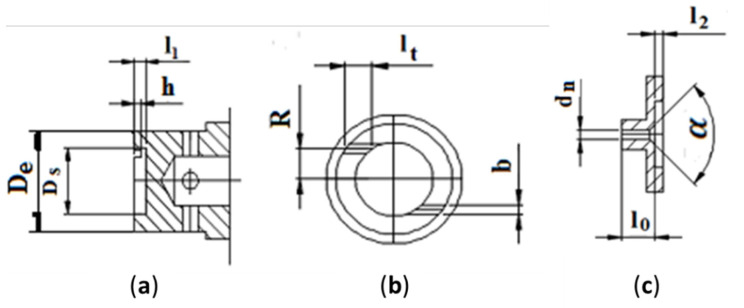
The geometrical parameters of a pressure swirl atomizer: (**a**) swirl chamber of a mechanical atomizer, (**b**) front view of the swirl chamber, (**c**) nozzle of the swirl chamber.

The tangential ducts of the swirl chamber in the mechanical atomizer take the form of two grooves, of width *b* and the depth *h* (Figure 3a,b); the water is subsequently let out via the channel with diameter *d_n_* (Figure 3c) in the nozzle (Figure 2a—3).

To obtain an optimal geometry for the mechanical atomizer, the diameter of the swirl chamber *D_s_* was set to be 4 mm, the width of the grooves (equal to 2) *b* = 0.5 mm, depth *h* = 0.4 mm, the swirl radius *R* = 1.65 mm, and the outer diameter *D_e_* = 6 mm (Figure 3). The height of the swirl chamber was set at *L_s_* = *l_1_* + *l_2_* = 2 mm. The nozzle exit channel was set to have diameters *d_n_* = 0.6 and 0.8 mm, and length *l_0_* = 2 mm. At these dimensions, the parameters were *L_s_/D_s_* = 0.5; *l_0_/d_n_* = 3.3 (*d_n_* = 0.6 mm) and 2.5 (*d_n_* = 0.8 mm); *l_t_/b* ≈ 2. The convergence angle of the swirl chamber was α = 90°. In line with the derivation of geometrical dimensions of Equation (1), it was determined that *A* = 3.88, with a nozzle exit channel *d_n_* = 0.6 mm, and *A* = 5.18 with *d_n_* = 0.8 mm. The air swirl chamber was adjusted to have diameter *D_a_* = 7 mm and tangential channels (*n* = 4) *d_t_* = 1.8, 2 and 2.4 mm and *R_a_* = 2.2 mm (Figure 2b). The geometrical characteristics *A_a_* of an air swirl chamber are as follows: *A_a_* = 2.4 at *d_t_* = 1.8 mm; *A_a_* = 1.9 at *d_t_* = 2 mm; and *A_a_* = 1.34 at *d_t_* = 2.4 mm. 

### 2.3. Experimental Setup and Methodology

Atomization parameters were measured by the TSI application software (TSI Inc., Co, Shoreview, MN, USA), which is part of the TSI phase Doppler particle analyzer/laser Doppler velocimeter (PDPA/LDV) system. PDPA is an optical technique based on an LDV, which allows simultaneous measurement of sizes and velocities of spherical particles by use of a coherent laser beam. The basics of the TSI PDPA/LDV measurement system are described elsewhere [44,45].

**Figure 4:**
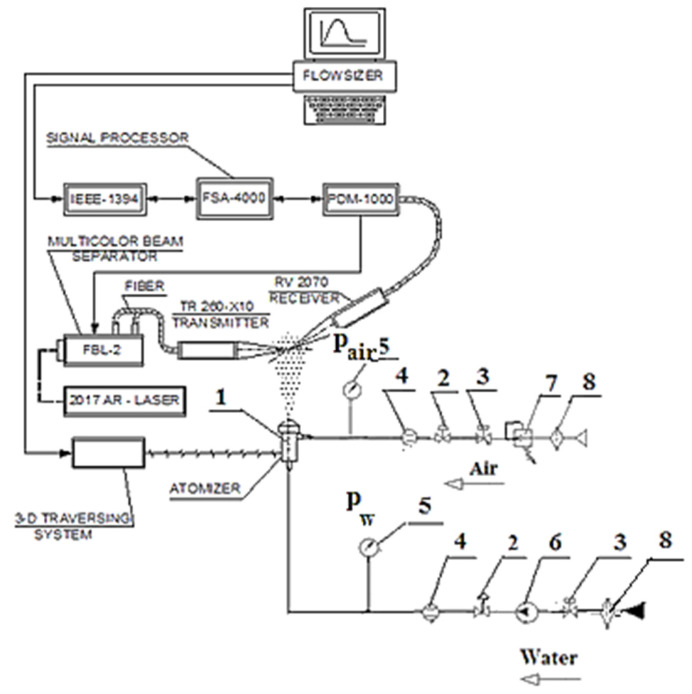
A scheme of the test rig: 1—atomizer, 2—control valve, 3—shut-off valve, 4—flow meter, 5—pressure gauge, 6—water pump, 7—air pressure regulator, 8—filter.

The experimental set-up is presented in Figure 4. Measurements of spray characteristics were carried out 50 mm downstream from the atomizer tip with 5 mm steps perpendicular to the spray cone diameters. According to Valencia-Bejarano et al., a distance of 50 mm is sufficient to form droplets but not sufficient to affect droplet size by coalescence [46]. The system automatically measured the drop diameters, after entering the measurement volume, which is less than 10^−3^ mm^3^. The spray angle was measured based on the liquid flux distribution and digital photographs. 

The atomizer’s water and air flow rates were obtained in the tests, as well as histograms for the drop diameter distribution under inlet water pressure values of *P_w_* = 2–7 bar in the experimental mechanical atomizer. During the experiment with the two-phase atomizer, the presented characteristics were obtained with a water pressure of 2–3.5 bar and air supply pressures of 0.35, 0.7 and 1 bar.

The resulting data were used for calculation of the mean *D*_32_ drop diameter, defined by the following relation:

(2) $$D_{32}=\frac{\sum_{i=1}^{N}D_{i}^{3}}{\sum_{i=1}^{N}D_{i}^{2}}$$

Here, *D*_32_ is the Sauter mean diameter, which gives the mean diameter value in terms of the volume/surface ratio. 

## 3. Results and Discussion

### 3.1. The Mechanical Atomizer

The mechanical atomizer was designed to have optimal geometrical parameters, taking into account data from numerous publications regarding the minimal value of the flow coefficient and thus providing a high-quality liquid spray. Using the test results the flow rate coefficient (*C_d_*) was calculated by Equation (3) [39]:

(3) $$C_{d}=\frac{Q_{w}}{\pi r_{n}^{2}\sqrt{\frac{2}{\rho_{w}}P_{w}}}$$

where *Q_w_* is water volume flow rate (m^3^/s), *P_w_* is the inlet water pressure (Pa), *ρ_w_* is the water density (kg/m^3^), and *r_n_* is the radius of the discharge nozzle (m). In the atomizer test, under an inlet water pressure of *P_w_* = 5 bar, a flow rate of 3.5 × 10^−3^ kg/s was obtained; the flow rate coefficient was *C_d_* = 0.22. This value of the coefficient was compared to the value that was estimated empirically by Equation (4) [27]:

(4) $$C_{d}=0.35\cdot {\left(\frac{F_{t}}{D_{s}\cdot d_{n}}\right)}^{0.5}\cdot {\left(\frac{F_{t}}{d_{n}}\right)}^{0.25}$$

where *F_t_* is the inlet tangential cross sectional area (m^2^), *d_n_* is the exit nozzle of the swirl chamber (m) and *D_s_* is the diameter of the swirl chamber (m). The received flow coefficient *C_d_* from Equation (4) is equal to 0.244. The experimental value of *C_d_* is lower than the empirical value, which attests to the optimal choice of the atomizer’s geometrical parameters (with minimal hydrodynamic losses and the high possible ratio between the tangential and axial velocities). 

Atomization can be represented by the widely used Weber number (*We*), which estimates when the liquid jet is likely to break up (Equation (5)).

(5) $$We=\frac{\rho_{aU_{r}d_{n}}}{\sigma }$$

where *U_r_* is the relative velocity between the liquid jet and ambient (m/s), *ρ_a_* is the air density (kg/m^3^), *d_n_* is the exit nozzle of the swirl chamber (m), and *σ* is the surface tension (kg/s^2^). Taking into account the value *U_r_* equal to 31.6 m/s at *P_w_* = 5 bar, *We* = 14 is considered as favorable condition for the liquid jet break-up (*We* must be≫1. The inlet ports Reynolds number is defined as:

(6) $$Re=\frac{U_{in}\cdot 2r_{in}}{\nu }$$

where *U_in_* is the water velocity in the tangential ports (m/s), *r_in_* is the radius of the tangential ports (m), and ν is the water viscosity (kg/m·s).

(7) $$U_{}=\frac{Q_{w}}{4\pi \cdot r_{in}^{2}}$$

Changing pressure in the diapason of 2–7 bar, the Reynolds number is in the range of 1.4 × 10^4^–2.6 × 10^4^. For the given Reynolds numbers, the length of the input ports (*l_t_/d_t_* = 2) is sufficient to completely fill it [38]. 

**Figure 5:**
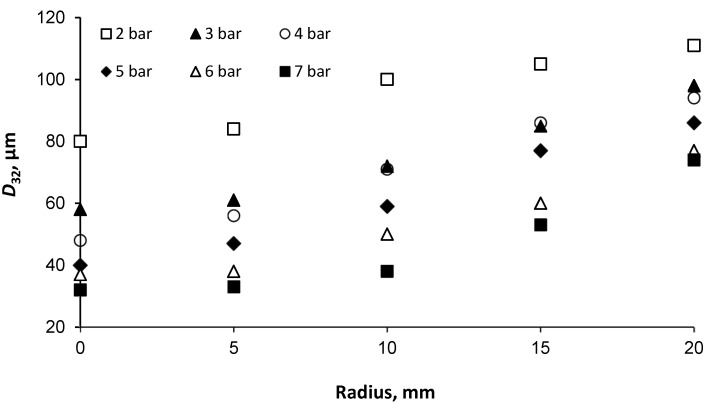
Distribution of *D_32_* drop diameters over the radius of the spray torch.

The distribution of drop diameters of the mechanical atomizer are presented in Figure 5. At a pressure of 2 bar, the *D*_32_ of the water drops on the torch axis was 80 μm. As the distance from the axis of the torch to its periphery (the radius) was increased, the drop diameter rose to 110 μm. Under increased water pressure the drop diameter in the paraxial zone of the torch decreased, as has been noted by many researchers, and was 58 μm at *P_w_* = 3 bar, 48 μm at *P_w_* = 4 bar, 40 μm at *P_w_* = 5 bar and 37–32 μm at *P_w_* = 6–7 bar. But in the peripheral zone the decrease in the atomized drop diameter as the pressure increases was less significant. 

Experiments that were carried out with a Danfoss company atomizer at a water pressure of *P_w_* = 8 bar yielded spray particle diameters of *D_32_* equal to 60 µm with radius *R* = 0, and 115 µm with *R* = 10 mm, while at *P_w_* = 13 bar the spray particle diameters were 40 and 50 µm, respectively. Rashad et al. investigated the influence of certain geometric ratios of swirl chamber parameters on drop diameters. The authors obtained *D*_32_ equal to 60–70 µm at the supply pressure of *P_w_* = 8 bar, and 50–60 µm at *P_w_* =12 bar [30]. It must be noted that, according to Xue et al., 58% of the pressure drop converts into the kinetic energy of the rotational motion in the swirl chamber, an energy loss which includes both hydraulic loss and friction loss [40]. Consequently, optimization of the atomizer swirl chamber geometry can significantly improve the quality of the spray. In addition, the *D*_32_ drops of the developed mechanical atomizer were calculated using empirical Equation (8) [27] and Equation (9) [47]:

(8) $$D_{32}=2.25\sigma^{0.25}\mu_{l}^{0.25}m_{l}^{0.25}\rho_{g}^{-0.25}\Delta P_{l}^{-0.5}$$

(9) $$D_{32}=4.4\sigma^{0.6}\mu_{l}^{0.16}\rho_{l}^{-0.16}m_{l}^{0.22}\Delta P_{l}^{-0.43}$$

For example, at *P_w_* = 5 bar the *D*_32_ according to Equations (8) and (9) were expected to be 64 and 82 µm, respectively. The difference between the empirical and measured results is due to several reasons. First is the limited region in which the PDPA measurements were taken. Droplets can experience secondary breakup and produce smaller droplets further downstream, beyond the range of the physical apparatus at our disposal. Additionally, since the physical phenomena involved in atomization processes are not fully understood, the empirical correlations cannot fully represent the physical principles determining the droplet formation process. Finally, these empirical models were developed based on fuel spray measurements in high pressure conditions such as engine combustion, whereas in our study the sprays operate at a much lower pressure, thus some deviation is inevitable. It must be noted that the *D*_32_ drops in the pressure range of 2–5 bar on the torch axis decrease by 50%, but in the range of 6–7 bar they decrease only by 13.5%. Therefore, when using an atomizer with the optimal geometrical dimensions as we have stated, the fluid supply pressure should be limited to 5 bar. 

**Figure 6:**
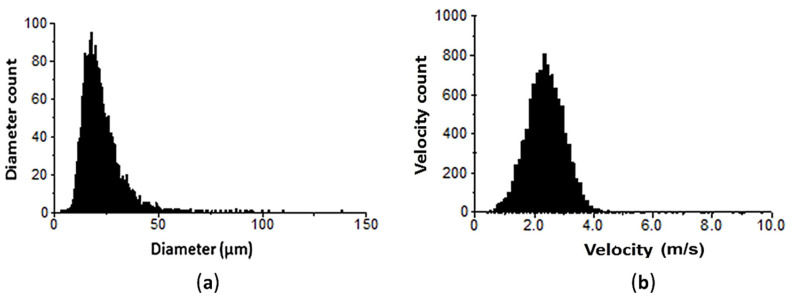
Diameter (**a**) and velocity (**b**) histograms at *P_w_* = 4 bar, *R* = 0 mm.

The standard histograms for the distribution of drop diameters and velocities in the paraxial zone (*R* = 0) are presented in Figure 6. As can be seen from the data obtained, the peak of the histogram occurs within 15–25 μm. This is in accordance with the finding of Chang et al. [48], who reported that the smaller drops are mostly confined to the core regions of the spray. The larger drops, more than 40 μm, occupy the peripheral part of the spray. 

**Figure 7:**
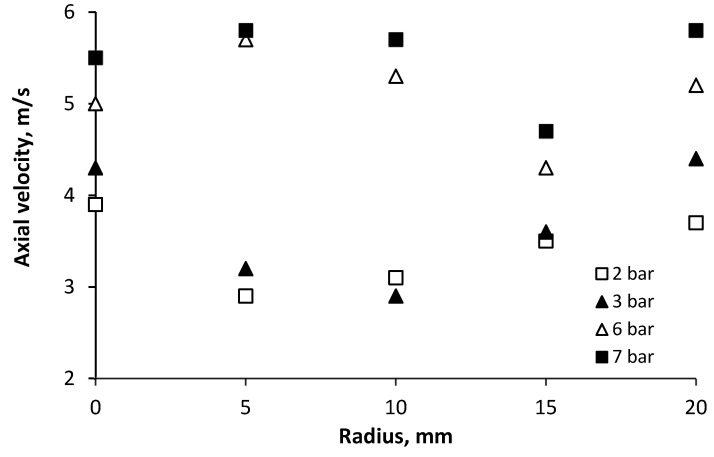
Axial droplet velocity vs radius for different water pressures.

The droplets’ axial velocity distribution helps to facilitate the expansion of the spray, and the diffusion to the ambient air, which is more intense in the downstream region of the spray where it promotes turbulent mixing. In Figure 7 the change in axial drop velocity is shown for pressures of 2 and 3 bar, where bigger drop diameters are produced, and for pressures 6 and 7 bar, where smaller drop diameters are produced.

As can be seen from these measurements, in the cross section at 50 mm downstream from the nozzle, most droplets have a velocity ranging from 3 m/s to 5.5 m/s. This figure also illustrates that droplets of different sizes at various locations, can have the same velocities depending on their trajectory. However, most of them are in a speed range that gives them sufficient momentum to penetrate the surrounding medium. A higher water supply pressure, together with smaller droplets (pressure 6–7 bar), yields higher velocities because of their larger drag-to-momentum ratio. The initial discrepancies between the velocities of various sizes drops are preserved over the entire jet torch cross section.

**Figure 8:**
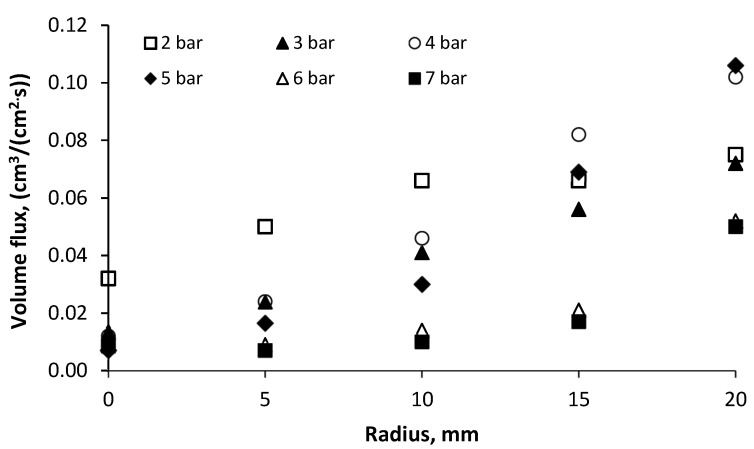
Volume flux vs radius for different water pressure.

The generated spray of a cone-shaped pattern of volume flux is illustrated in Figure 8, which is characterized by the high concentration of water drops at the edges of the spray cone and low concentration in the central part of the spray. 

**Figure 9:**
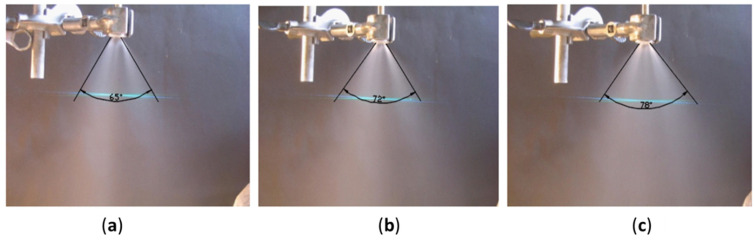
The spay torch angle (*β*) of the mechanical atomizer at varying water pressures: (**a**) 3 bar, (**b**) 4 bar, (**c**) 5 bar.

It is essential to know the spatially distributed flux density of a mechanical atomizer for the design of a spray system. These measurements are complementary to the drop size, the coefficient of discharge and the spray cone angle. From the obtained data shown in Figure 5, the smallest drops are located in the central part of the spray cone. These small-sized drops have a large surface/volume ratio, and evaporate more quickly than the larger ones, which is why the central part of the spray has a low drop concentration. The volume flux in the central part is at a minimum and decreases with increased water pressure, which yields the smallest drop sizes. For example, the volume flux in the central part was 0.007 cm^3^/(cm^2^·s) at *P_w_* = 5 bar, and 0.032 cm^3^/(cm^2^·s) at *P_w_* = 2 bar. Because large drops evaporate with relative difficulty and hence accumulate at the edge of the spray, the bigger flux volume is concentrated on the jet periphery, as is shown in Figure 8. In the peripheral region the jet volume flux is equal to 0.075 cm^3^/(cm^2^·s) at *P_w_* = 2 bar, and 0.106 cm^3^/(cm^2^·s) at *P_w_* = 5 bar. Additionally, the spray angle slightly increases with increased water pressure, as seen in Figure 9, and is in the range *β* = 65°–78°.

### 3.2. The Air-Assisted Atomizer

**Figure 10:**
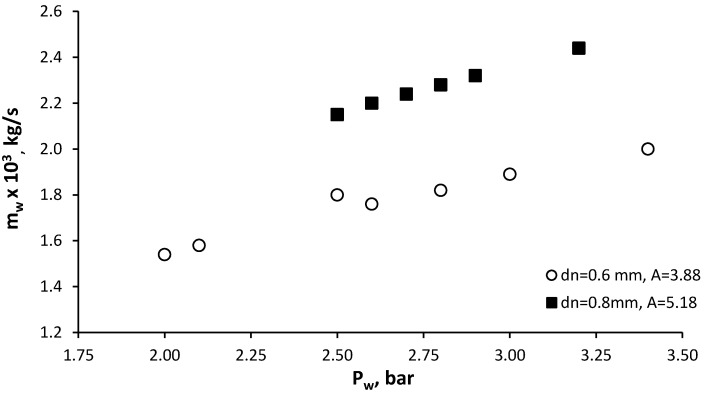
Water flow rate characteristic of the mechanical atomizer.

To obtain small diameters of liquid droplets at low supply pressures, a gas flow is introduced into the atomizer. In our studies, a liquid atomizer with an optimal geometry was installed at the first stage for liquid spraying. At the second stage the effluent conical jet interacts with the air flow which is introduced through the tangential channels of the swirl chamber. The test results of the mechanical atomizer that provides preliminary water spray in the air-assisted atomizer with different exit nozzle diameters *d_n_* = 0.6 mm and 0.8 mm are shown in Figure 10.

The flow rate coefficient *C_d_* of the mechanical atomizer, calculated by Equation (3), was equal to 0.27 and 0.19 with the nozzle diameters 0.6 mm and 0.8 mm, respectively. With the air flow, using a channel diameter of 0.6 mm, the value of A was higher than the value was without the air flow (3.88 vs. 3.2). The calculated value of *C_d_* in the presence of air flow was actually higher, though we expected it to be lower. This is explained by a significantly larger value *l_0_/d_n_* (3.3 and 2.5), leading to an increase in hydraulic losses, which corresponds to the data obtained in [32,38]. The Reynolds number (Equation (6)) for flow from the exit nozzle at operating parameters was in the range 8.5 × 10^3^–1.1 × 10^4^ for diameter *d_n_* = 0.6 mm and 1.1 × 10^4^–1.5 × 10^4^ for *d_n_* = 0.8 mm. The values of coefficient *C_d_* for these test variants were also determined in accordance to Equation (4) and found *C_d_* = 0.23 for *d_n_* = 0.6 mm, *C_d_* = 0.185 for *d_n_* = 0.8 mm, which approximates to the experimental data. 

**Figure 11:**
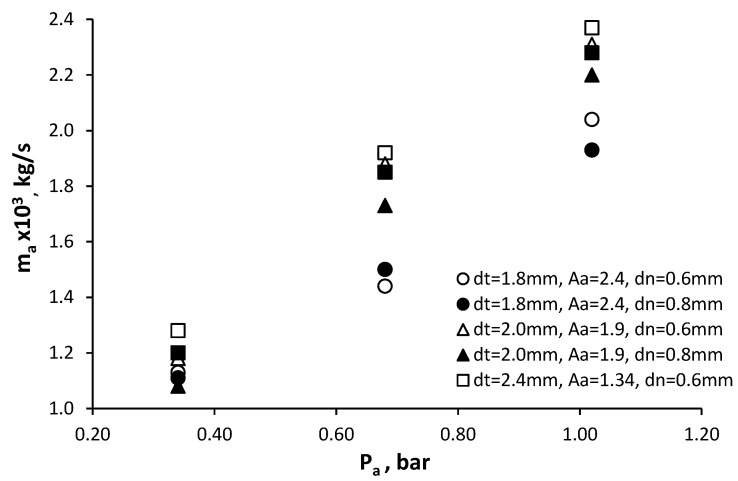
Air flow rate characteristics of the swirl chamber.

Air flow rate characteristics of the swirl chamber are shown in Figure 11.

The test results show that the air flow rate through the swirl chamber is determined not only by its supply pressure and the diameter of the tangential channels, but also by the operating parameters of the mechanical atomizer. This can be explained by the influence of the torch angle of the liquid jet and its energy, which, when interacting with the air flow at the outlet of the tangential channels, has a certain resistance to its flow. For example, at *P_a_* = 1 bar and in a mechanical atomizer with a diameter of the exit nozzle of *d_n_* = 0.6 mm at *P_w_* = 2.5 bar, the air flow rate was 2.2 × 10^−3^ kg/s (*d_t_* = 1.8 mm). Using these operating conditions for the air swirl chamber but with mechanical atomizer parameters set at *d_n_* = 0.8 mm and *P_w_* = 3.2 bar, the air flow rate was 1.95 × 10^−3^ kg/s (−11.5%). At *d_t_* = 2 mm, *P_a_* = 0.7 bar and for a mechanical atomizer with *d_n_* = 0.6 mm, water supply pressure *P_w_* = 2.6 bar, the air flow rate was 1.88 × 10^−3^ kg/s, but when the nozzle was set to *d_n_* = 0.8 mm and *P_w_* = 2.7 bar the air flow rate was 1.73 × 10^−3^ kg/s (−8%). As can be seen from Figure 11, increasing the diameter of the nozzle *d_n_* reduces the air flow rate. This corresponds to the influence of the liquid jet torch angle which is determined by the geometrical characteristic *A*. According to theory [39], the torch angle *β* increases with increasing the characteristic *A*:

(10) $$tg\beta /2=\frac{2C_{d}\cdot A}{\sqrt{{\left(1+\frac{r_{\varphi }}{d_{n}/2}\right)}^{2}-4C_{d}^{2}A^{2}}}$$

where *r_φ_* is a cavity zone radius (mm) and equal $r_{\varphi }=d_{n}/2\sqrt{1-\varphi_{n}}$.

The $\varphi_{n}$ is determined by:

(11) $$A=\frac{\left(1-\varphi_{n}\right)\sqrt{2}}{\varphi_{n}\sqrt{\varphi_{n}}}$$

This is in accordance with the calculation for amechanical atomizer with *d_n_* = 0.6 mm β/2 = 48°, with *d_n_* = 0.8mm β/2 = 62°. Thus, an increase in the jet torch angle at the exit of the mechanical atomizer reduces the air flow rate. 

**Figure 12:**
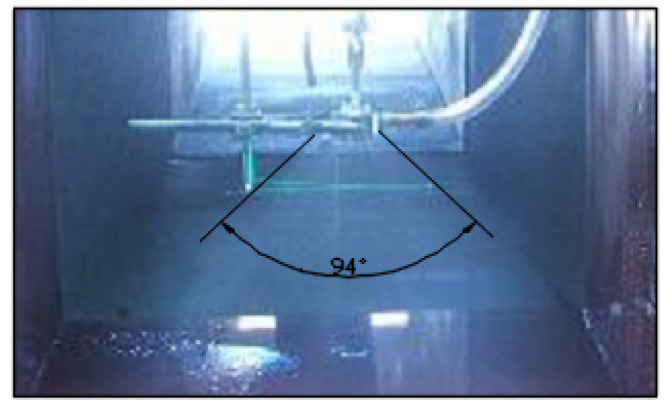
The spray torch angle of an air-assisted atomizer.

Introducing swirling air flow at the exit of the mechanical atomizer affects the radial velocity component. This effect should lead to a widening of the spray cone angle and have a significant influence on the drop size. The spay torch angle of the air-assisted atomizer increases to *β* = 90°–95° and is shown in Figure 12.

**Figure 13:**
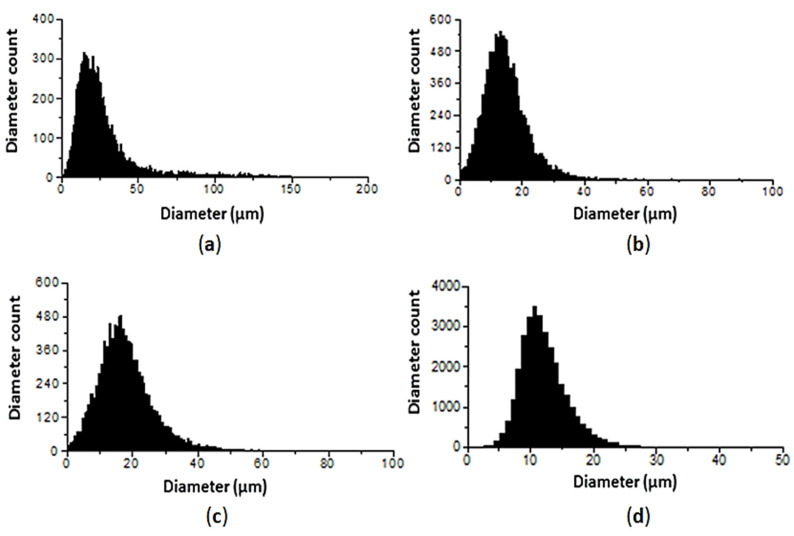
The influence of operation conditions on the atomization quality of the atomizer assembly. (**a**) *d_t_* = 2.0 mm, *d_n_* = 0.6 mm, *ALR* = 0.69, *P_w_* = 2.5 bar, *P_a_* = 0.35 bar; (**b**) *d_t_* = 2.0 mm, *d_n_* = 0.6 mm, *ALR* = 1.19, *P_w_* = 3 bar, *P_a_* = 1 bar; (**c**) *d_t_* = 2.4 mm, *d_n_* = 0.8 mm, *ALR* = 0.67, *P_w_* = 2.5 bar, *P_a_* = 0.35 bar; (**d**) *d_t_* = 2.4 mm, *d_n_* = 0.8 mm, *ALR* = 0.97, *P_w_* = 2.9 bar, *P_a_* = 1 bar.

One of the parameters determining spray quality in the air-assisted atomizer is the air to water flow ratio (ALR). The distribution drop sizes of some versions of the atomizer assembly are given in Figure 13.

As can be seen from Figure 13, the introduction of a swirling air flow at the exit of the jet from the mechanical atomizer significantly reduces the diameter of the droplets, even at low supply pressures for water and air. Moreover, with an increase in ALR, the diameter of the droplets decreases to 20–30 µm.

**Figure 14:**
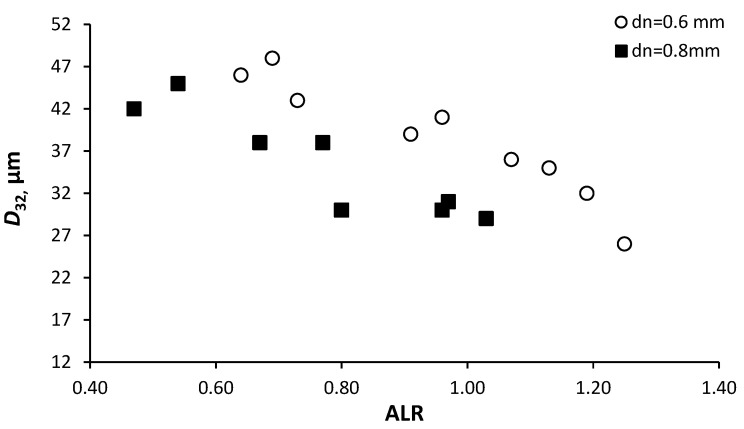
Dependence of *D*_32_ drop diameters on ALR.

The dependence of *D*_32_ water drop diameters on the ALR of an operating air swirl chamber that has various tangential channels and that uses a mechanical atomizer equipped with various exit nozzles is shown in Figure 14. 

Installing the air swirl chamber substantially decreases drop sizes, as these are affected by the energy of the air. For all ranges of values of ALR = 0.47–1.25 and *P_w_* = 2–3.4 bar, the *D*_32_ drops were in the range of 25–48 μm respectively (using only the mechanical atomizer, the *D*_32_ ranged from 58 to 110 μm at *P_w_* = 2–3 bar). The test results show that the geometrical characteristic of the air swirl chamber (*A*_a_ = 1.34–2.4) does not exert a considerable influence upon atomization quality. At the same time, the geometrical characteristic *A* of the mechanical atomizer does influence the results via the quality of the preliminary atomization. Thus, a mechanical atomizer with *A* = 5.18 (*d_n_* = 0.8 mm) has all the necessary prerequisites for producing more finely dispersed water atomization. For example, the *D*_32_ drop diameters equal to 35 μm at ALR = 1.13 were obtained in the test with the air swirl chamber when the tangential ducts were set at *d_t_* = 1.8 mm and the nozzle diameter at 0.6 mm; but with the nozzle diameter set at *d_n_* = 0.8 mm, results were *D*_32_ = 30 μm at ALR = 0.8 (Figure 14). In the test with the air swirl chamber with tangential channels diameter *d_t_* = 2 mm and nozzle *d_n_* = 0.6 mm, the *D*_32_ drop diameter was equal to 48 μm at ALR = 0.69, while with the nozzle set at *d_n_* = 0.8 mm, *D*_32_ = 45 μm was obtained at ALR = 0.54. It must be noted that in the test of the air-assisted atomizer in [49], *D*_32_ of 40–35 μm was obtained at ALR = 5–9.

Finally, it should be noted that an air supply pressure of over 1 bar does not have a significant influence upon the quality of atomization. This two-phase atomizer provides small diameters of spray droplets at significantly lower air supply pressures than two-phase atomizers of well-known companies. Since the compressed air supply pressure has the main effect on the energy consumption compared to liquid pressure, the use of this atomizer will reduce the energy cost. Consequently, we are able to recommend an air and water flow rates ratio setting of ALR ≈ 1.

## 4. Conclusion

The possibility of improving liquid atomization by using a mechanical atomizer and introducing an air-assistance component has been investigated. We have shown that with optimization of the atomizer’s geometric parameters, it is possible to obtain drop diameters of *D*_32_ = 80 μm at a water supply pressure of *P_w_* = 2 bar, and *D*_32_ ≈ 40 μm at *P_w_* = 4–5 bar. In this design variant, further increases in pressure beyond 5 bar do not, practically speaking, influence the atomization quality.

This high-quality atomization was obtained with a low consumption of energy by using an air-assisted component, with the mechanical atomizer optimized for its geometric parameters installed as the first stage for preliminary spray of the water, and with a swirl air flow interaction supplied at low pressure as the second stage. Results obtained from the experimental test produced drop diameters of *D*_32_ = 20–30 µm at the water pressures of 2.5–3 bar and air-supply pressures of 0.35–1 bar, and at ALR ≈ 1. The drop diameters decrease if the ALR is increased. Further increasing air-supply pressure beyond 1 bar does not result in significant decreases in water drop diameters. The supply of an air flow increases the spray torch angle to 90°–95°. 

From this study’s results, it can be seen that the micro-droplet atomizer works in wide range of flow rate and low inlet pressure of liquid and gas. This suits for development of liquid fuels micro-turbine and other generator systems.
